# Drought Induced Signaling in Rice: Delineating Canonical and Non-canonical Pathways

**DOI:** 10.3389/fchem.2018.00264

**Published:** 2018-09-12

**Authors:** Prasanta K. Dash, Rhitu Rai, Vandna Rai, Surendranath Pasupalak

**Affiliations:** ^1^ICAR-NRC on Plant Biotechnology, Pusa Institute, New Delhi, India; ^2^Orissa Univesity of Agriculture and Technology, Bhubaneswar, India

**Keywords:** drought, plant growth, crops, rice, abiotic stress, lipid signaling

## Abstract

Drought induced stress is often a bottleneck of agricultural crop production. Invariably, field crops across all agro-ecological regions succumb to it with an yield penalty. Drought massively affects the growth and harvestable yield in crops and has become an imminent problem necessitating breeding of tolerant crops. It induces myriad changes of biochemical, molecular, and physiological nature that manifest into aberrant plant morphology. The response to drought in plants incites a signaling cascade that involves perception and translation of drought signal leading to concomitant modulation of gene expression and *de novo* osmolyte synthesis. The intricate patterns of expression of these genes vary from early induction to late responsive genes. While one class of genes codes for products imparting osmotolerance and protection to plants, the second class predominantly modulates target gene expression by an intricate signal transduction mechanism. This review summarizes both canonical and non-canonical cascades of drought stress response in plants, delineating the mechanism in rice (*Oryza sativa*) and emphasizes hydropenia induced lipid signaling.

## Introduction

Altered physiological conditions disrupt cellular homeostasis and orchestrate stress in plants. Invariably, plants during their growth period are exposed to multiple stresses such as drought, shading (low light intensity), low temperature, salinity, flooding, heat, oxidative stress, and heavy metal toxicity (Shivakumara et al., 2017; Shivaraj et al., 2017). All these stresses, individually or in combination eventually hamper productivity of the crops (Fang and Xiong, 2015; Joshi et al., 2016; Gupta et al., 2017). Amongst all, drought i.e., hydropenia is the most devastating environmental stress (Gaspar et al., 2002) and impacts multiple morphological changes that are visible in all “Phenological stages of plant/crop growth” (Zhang et al., 2017). It decreases crop stand in field (Lambers et al., 2008) and reduces harvestable yield and economic harvest in crops (Thirunavukkarasu et al., 2017; Van Gioi et al., 2017). It is estimated that drought will impact 30% global loss of crop yield by 2025 (Zhang, 2011). With climate change looming large, water deficit has become a cardinal issue of agriculture as climate models have predicted an increase in severity and frequency of drought (Walter et al., 2011; IPCC, 2012). Additionally, the growing water scarcity/mis-management of the available water is a major threat to sustainable domestic, industrial, and agricultural development (Hamdy et al., 2003).

Edaphologically, drought in crops results from short-fall in the required precipitation leading to reduced available water in the soil. Additionally, dry atmospheric condition increases water loss from plants by evapo-transpiration. Aside the precipitations, drought depends on evapo-transpiration, soil water holding capacity, crop water requirements, and ability of plants to efficiently utilize available water (Toker et al., 2007). Multiple molecular (Pornsiriwong et al., 2017) and cellular responses (Comas et al., 2013; Chen et al., 2017; Pornsiriwong et al., 2017) become operative with on-set of drought in plants. Nevertheless, tolerence to drought amongst crops shows variation between and/or within the crops. Depending upon the exhibited symptoms, effect of drought on plants are classified as slight, moderate, severe, and very severe on the basis of relative water content (RWC) (Gigon et al., 2004). While plants are able to withstand slight stress by evoking tolerance mechanism, mild drought induces regulation of water loss and uptake in plants allowing maintenance of relative water content (RWC) with minimally altered photosynthetic capacity and quantum yield.

## Impact of drought on plant growth and yield

Drought hinders plant growth/development with commensurate reduction in accumulation of biomass. Farooq et al. (2009) and Li et al. (2009) identified that the consequences of drought in crop plants range from reduced (i) cell division and expansion, (ii) leaf size and stem elongation, (iii) perturbed water/nutrient relations and stomatal oscillations, and (iv) diminished water use efficiency (WUE) (Farooq et al., 2009; Li et al., 2009). With on-set of water deficit, abscisic acid (ABA) biosynthesis is stimulated in plants which reduces stomatal conductance and transpirational losses (Yamaguchi-Shinozaki and Shinozaki, 2006). Cell division and cell enlargement in drought stressed plants is negatively affected as water potential/cellular turgor is lost, and photosynthesis decreases (Farooq et al., 2009; Taiz and Zeiger, 2010). These massive physiological changes in plants reduce root, shoot, and flower fresh/dry weight (Liu et al., 2011) with maximum reduction in total leaf area (Farooq et al., 2010). It also affects crop phenology and induces early transition from the vegetative to the reproductive phase (Desclaux and Roumet, 1996) leading to altered crop growth cycle.

Hydropenia has pronounced negative effect on crop yield. Particularly, drought during silking stage in maize reduces total biomass accumulation by 37%. It also reduces yield by negatively affecting at grain-filling stage (reduction by 34%) and at maturity (by 21%) (Kamara et al., 2003). In rice, drought-induced physiological changes such as stomatal closure decreases intake of CO_2_ and eventually decreases photosynthesis (Flexas et al., 2005) due to reduction in carbon capture that imbalances the source and sink partitioning of photosynthets, reduces the phloem loading, reduces assimilate translocation and dry matter partitioning (Farooq et al., 2009). Additionally, photorespiration becomes operative which leads to decline in the carbon fixation in rice. In C_3_ plants, such as rice, Rubisco is the key enzyme in CO_2_ assimilation and acts either as carboxylase or as an oxygenase depending upon the internal concentration of CO_2_/O_2_ in leaf_._ At moderate water stress with closed stomata, Rubisco acts as an oxygenase as prevailing cellular O_2_ concentration is higher than CO_2_ concentration. This increase in photorespiration due to drought at the “Expense of carbon-fixation” (Ghannoum, 2008) leads to yield reduction in rice.

## Canonical mechanisms of drought resistance in plants

Plants evoke myriad morphological and biochemical adaptations at whole-plant and cellular-levels to ward off stresses of drought. Noteworthy, among them are the three canonical mechanisms such as (i) drought escape, (ii) drought avoidance, and (iii) drought resistance (Yamaguchi-Shinozaki and Shinozaki, 2006). Drought escape is the mechanism that invigorates plants to complete their life cycle before drought sets in, so that the seeds enter in to dormancy before the dry conditions prevail e.g., desert plants saving themselves from extinction. However, “Drought avoidance mechanism in plants involve maintaining high water status/cellular hydration” either by absorbing more water from soil or by reducing loss of water by transpiration. In contrast, drought tolerance is the ability of plants to continue normal cellular metabolism and growth activity at low water potential despite prevailing stress condition and/or ability to recover fast after stress. A crop is considered tolerant, only if it survives drought with minimal yield penalty. These plants maintain the cellular turgor through osmotic adjustment and protoplasmic resistance (Mitra, 2001) by accumulation of free proline (Munns, 2005).

## Molecular mechanism of drought tolerance: rice as a model

In response to drought, plants activate three main categories of genes that canonically modulate biochemical/physiological and/or molecular pathways (Dash et al., 2014). They are (1) genes involved in “protection of membranes; water and ion uptake/transport” imparting cellular tolerance (2) regulatory genes involved in signaling/transcriptional control, and (3) novel genes of unknown function reported to impart drought tolerence. Plants extrinsically perceive environmental stress and transfer the signal through cascades of molecules. These signaling molecules trigger the expression of specific genes leading to appropriate physiological/biochemical responses (Shinozaki and Yamaguchi-Shinozaki, 2007; Golldack et al., 2014; Hu and Xiong, 2014). A number of genes/transcription factors showing differential expression to drought have been identified in plants (Yamaguchi-Shinozaki and Shinozaki, 2006; Joshi et al., 2016). These are known to be involved in cellular responses such as “stress perception and transcriptional regulation” of drought responsive genes (Lata and Prasad, 2011). These genes code for “Protein kinases, phytohormones, transcription factors” (Lata et al., 2015), osmoprotectants and “late embryogenesis abundant (LEA)” proteins (Varshney et al., 2011; Golldack et al., 2014; Todaka et al., 2015; Sah et al., 2016) imparting tolerance to dehydration.

Perceived response to drought in plants is broadly categorized into ABA-dependent or ABA-independent pathway. In rice, ABA-Responsive cis-Elements (ABRE;PyACGTGG/TC) are enriched compared to *Arabidsopsis* and soybean (Maruyama et al., 2011) and in response to drought, ABA concentration dramatically increases in vegetative parts. Increased ABA triggers (i) stomatal closure, (ii) stress proteins and metabolites accumulation (protect cells during stress), and (iii) H_2_O_2_ accumulation in guard cells that signals reduction in water loss from the plant (Mustilli et al., 2002; Kwak et al., 2003; Wang and Song, 2008). The ABA independent pathway, elucidated earlier, involves H_2_O_2_ mediated stomatal closure in rice (Huang et al., 2009).

The signaling mechanism to drought in plants involves sensing and relaying of dehydration signal from plasma membrane to the nucleus (Sanders et al., 1999; Ramanjulu and Bartels, 2002). This is accomplished through several protein phosphorylation mechanisms involving kinases viz. the mitogen activated protein kinases (MAPKs) and receptor-like kinases (RLKs) (Das and Pandey, 2010; Tena et al., 2011; Seybold et al., 2014). In rice, “*DROUGHT-HYPERSENSITIVE MUTANT1* (*DSM1*)”—a protein kinase—scavenges the reactive oxygen species (ROS) produced under drought stress. The *dsm1* mutants are hypersensitive to drought during seedling and panicle development stage (Ning et al., 2010). The stress-responsive RLK genes such as *stress induced protein kinase 1* (*OsSIK1*), growth under drought kinase (*GUDK*) were found to be induced by drought stress in rice. While, rice overexpressing *OsSIK1* showed tolerance to drought (Ouyang et al., 2010); GUDK phosphorylates *apetala 2/ERF37* (*OsAP37*) that activates stress-regulated genes (Ramegowda et al., 2014) in rice.

Several, transcription factors (TFs) regulating hydropenia signaling in rice has also been elucidated. Most of these TFs bind to cis-regulatory elements and belong to “AP2/ERF, bZIP, NAC, MYB, WRKY, bHLH, NF-Y, and CAMTA” families (Umezawa et al., 2006; Licausi et al., 2013; Castilhos et al., 2014; Shao et al., 2015). Over-expression of these TFs in rice showed increased ability of plant to withstand drought. Notably, *DREB* (dehydration-responsive element-binding protein) transcription factors act as key players in ABA independent pathway of drought tolerance. Among *DREB*s, *DREB1/CBF*, and *DREB2* are involved in drought stress (Srivasta et al., 2010; Nakashima et al., 2014). The transgenic rice plants expressing *DREB1A* yield more compared to the non-transgenic plants (Datta et al., 2012) under drought condition. Similarly, over-expression of NAC transcription factors (NAP and ONAC022) “reduce rate of water loss and transpiration, decrease number of open stomata and increase proline content” in rice (Hong et al., 2016). Nevertheless, at vegetative stage, they impart “enhanced tolerance to high salinity, drought, and cold” while increase yield despite drought in flowering stage (Liang et al., 2014).

Besides drought responsive elements, proteins have been identifed in hydropenia (Goyal et al., 2005). Accumulation of LEA have been detected in seeds as well as in vegetative tissues (Ingram and Bartels, 1996; He et al., 2012; Liu et al., 2013). Expression of LEA protein encoding genes, *OsEM1* and *OsLEA3-1* enhances tolerance of rice under water deficit (Xiao et al., 2007; Yu et al., 2016). Similarly, high cuticular wax in many crops imparts tolerance to drought (Xue et al., 2017). Crops having more cuticular wax than reduced/ non-waxy crops show drought-tolerance and higher yield (Zhou L. et al., 2013; Guo et al., 2016) due to strong correlation between the wax content and WUE (Zhu and Xiong, 2013). Recently, *waxy crystal-spare leaf 1* (*OsWSL1*) is reported to be involved in cuticular wax accumulation in rice (Yu et al., 2008) and *grain lenght 1-6* (*OsGL1-6*) has been identified to synthesize fatty aldehyde decarbonylase required for formation of wax in epidermis and in vascular bundles (Zhou L. et al., 2013). Mutants defective in *OsGL1* are sensitive to drought as they accumulate less cuticular wax (Mao et al., 2012). *DEEPER ROOTING 1* mutant (*DRO1*) governing root architecture and drought stress have been identified in rice. *DRO1* controls cell elongation at the root tip and changes the angle of root growth to downward direction (Uga et al., 2013) to fetch more water for growth.

Hydropenia induced abscisic acid (ABA) production also induces *de novo* expression of both structural and functional genes. Yamaguchi-Shinozaki and Shinozaki (2006) proposed operation of two pathways; (i) ABA-dependent pathway and (ii) ABA independent pathway. While the former pathway involves expression of genes “that may or may not require protein biosynthesis” the latter does not involve ABA for their induction. MYB and MYC transcription factors represent ABA dependent pathway while bZIP transcription factors don't require ABA synthesis and the target genes containing “abscisic acid response elements (ABREs) with core ACGT-containing G-box” (Chaves et al., 2003). The ABA independent pathway involves the “Water-deficit-inducible genes” that do not require ABA for their induction. The promoters of these genes contain a conserved “Dehydration responsive element (DRE)” and are induced by external stimuli (Yamaguchi-Shinozaki and Shinozaki, 2006).

## Lipid signaling in drought stress: the non-canonical mechanism

Besides activation of TFs/proteins; lipids are also involved in abiotic stress tolerance in plants (Okazaki and Saito, 2014; Hou et al., 2016). Seminal discoveries have elucidated lipid signaling in response to drought (Darwish et al., 2009; Golldack et al., 2014) in plants. Lipids such as wax, cutin, and suberin directly contribute to the alleviation of drought (Samuels et al., 2008) by reduction of cellular dehydration (Okazaki and Saito, 2014) and lipid metabolism (Gigon et al., 2004). It is reported (Kosma et al., 2009; Seo et al., 2011) that ABA treatment increases layers of these hydrocarbons in plants. While, overexpression of wax biosynthetic genes increases tolerance to drought (Yang et al., 2011; Luo et al., 2013; Zhou L. et al., 2013; Zhou M. et al., 2013), plants depleted of wax are less tolerant to drought (Qin et al., 2011; Seo et al., 2011; Mao et al., 2012; Zhu and Xiong, 2013). Lipid signaling in plants includes generation of “Inositol phosphate, lysophospholipids (LPLs), phosphatidic acid (PA), oxylipins, sphingolipids, diacylglycerol (DAG), free fatty acids (FFA), and N-acylethnolamine” that are generated from phospholipids (Munnik and Testerink, 2009; Saucedo-García et al., 2015). The mechanism involves generation of phosphatidic acid (PA) by rapid activation of phospholipase C (PLC) and phospholipase D (PLD) enzymes (Munnik et al., 1998, 2000; Pical et al., 1999). The enzyme PA kinase, by a attenuation mechanism, reduces phosphatidic acid (Munnik et al., 1996, 2000; Pical et al., 1999) to produce diacyl-glycerol pyrophosphate (DAGPP). Similarly, hyperosmotically stimulated cells change concentration of phosphatidylinositol phosphate, phosphatidylinositol 4,5-bisphosphate (Einspahr et al., 1988; Cho et al., 1993; Pical et al., 1999) and/or their novel isomers (Dove et al., 1997) to adjust to hydropenia.

**Figure 1:**
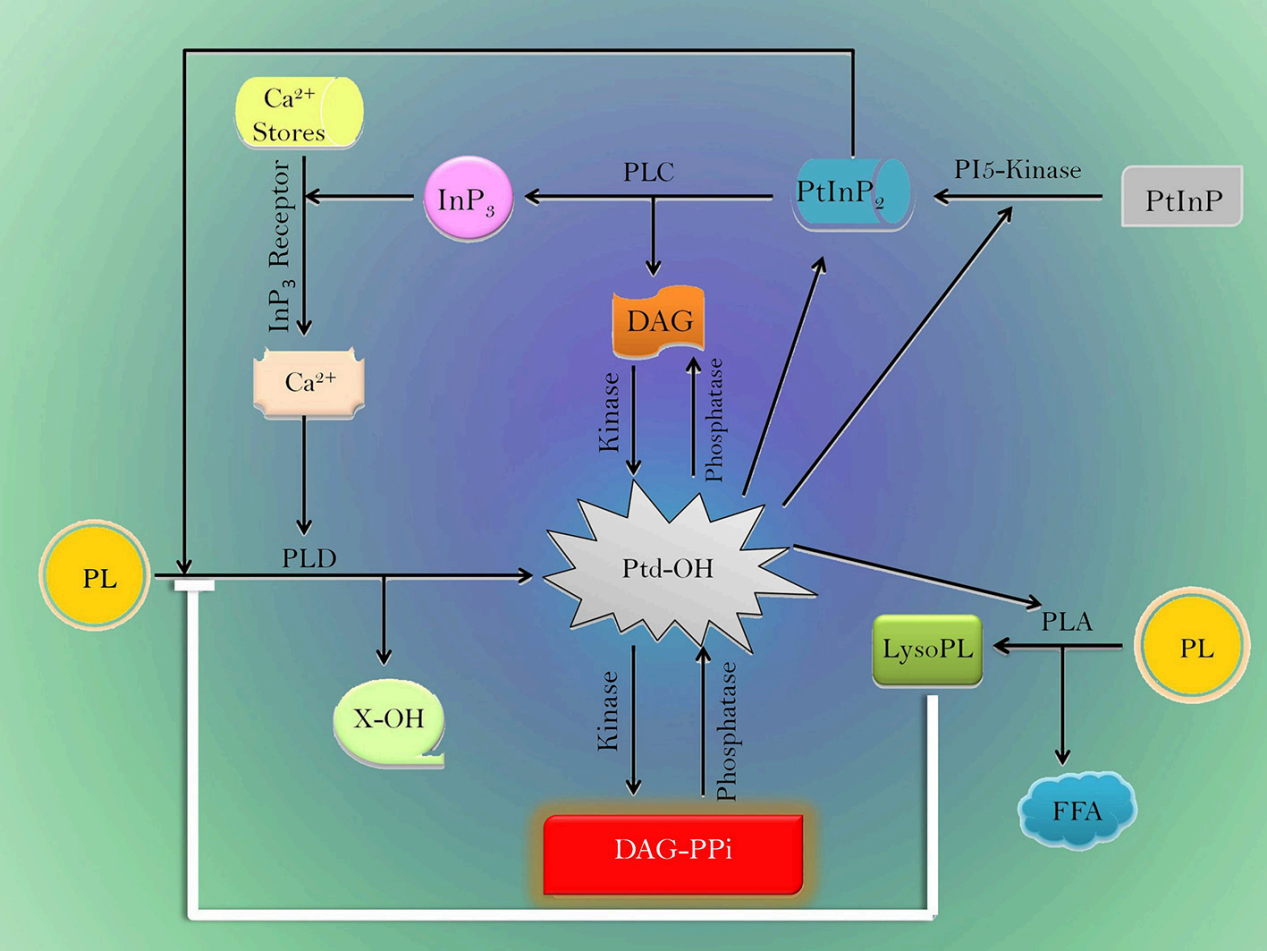
Model depicting the network of various phospholipases and lipid mediators during drought stress. Black line-induction, blue line-inhibition. PLA, Phospholipase A; PLC, Phospholipase C; PLD, Phospholipase D; DAG, Diacyl glycerol; DAG-PPi, Diacyl-glycerol pyrophosphate; PL, Phospholipid; Lyso PL, lysophospholipids; FFA, Free fatty acid; InP3, Inositol 1,4,5-trisphosphate; PI5-kinase, Phosphatidylinositol 4-phosophate 5-kinase; PtInP, Phosphatidylinositol monophosphate; PtInP2, Phosphatidylinositol 4,5-bisphosphate; PtdOH, Phosphatidic acid, and X-OH- Free head group.

The action of phospholipases and lipid intermediates depicting their role during drought is summarized in Figure 1. It reveals DAG and Inositol-3-phosphate are produced by PLC. InP3 increases the Ca^2+^ concentration in cytosol (Staxen et al., 1999). Consequently, Ca^2+^ and PtInP2 stimulate PLD (Wang, 2000; Zheng et al., 2000). The activated PLD generates phosphatidic acid from phospholipids. Cellular homeostasis of PtdOH is maintained by the opposing actions of kinsases and phosphatases that interconvert DAG, phosphatidic acid, and DAG-PPi in plants. Thus, the pool of phosphatidic acid acts as an important hub of lipid signaling/biosynthesis (Liscovitch et al., 2000). However, PLD directly does not alter activity of PLA but oxylipin synthesis is inferred to be activated by PLD (Wang, 2000). On the contrary, LysoPL produced by PLA directly inhibits PLD activity (Ryu et al., 1997) that maintains phospholipid homeostasis in plants.

With climate change looming large over modern intensive agriculture, frequency and severity of drought is predicted to increase. The erratic precipitation will cause large scale disruption in shallow rainfed rice agro-ecosystems leading to significant reduction in economic harvest. Thus, development of drought tolerant varieties by altering cellular homeostasis of lipids/proteins/carbohydrates is necessary for ensuring enhanced crop production in rainfed agro-ecological regions with unpredictable climatic conditions.

## Author contributions

PD and RR planned, collected information, and organized the manuscript. VR and SP provided critical input and edited the manuscript.

### Conflict of interest statement

The authors declare that the research was conducted in the absence of any commercial or financial relationships that could be construed as a potential conflict of interest. The reviewer MK and handling Editor declared their shared affiliation.
